# Supplementary material for: Potential for aerobic hydrocarbon oxidation in archaea
Source: Nat Commun. 2025 Oct 16;16:9188. doi: 10.1038/s41467-025-64223-2 (PMC12533140; doi:10.1038/s41467-025-64223-2)
Supplement: Supplementary file 1 — Supplementary Information [file 41467_2025_64223_MOESM1_ESM.pdf]

Supplementary Information

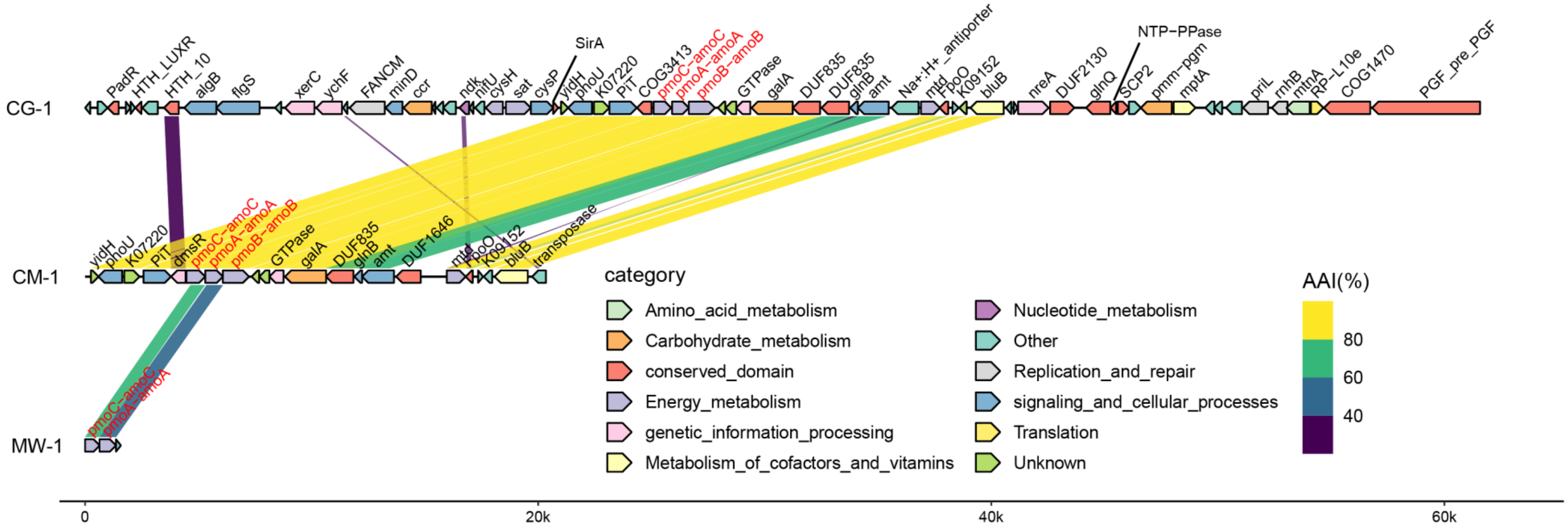

Supplementary Figure 1. Contigs encoding the CuMMO operons. Genes are labelled and coloured based on their KEGG classifications. AAI% between predicted proteins coloured. Scale is in base pairs.

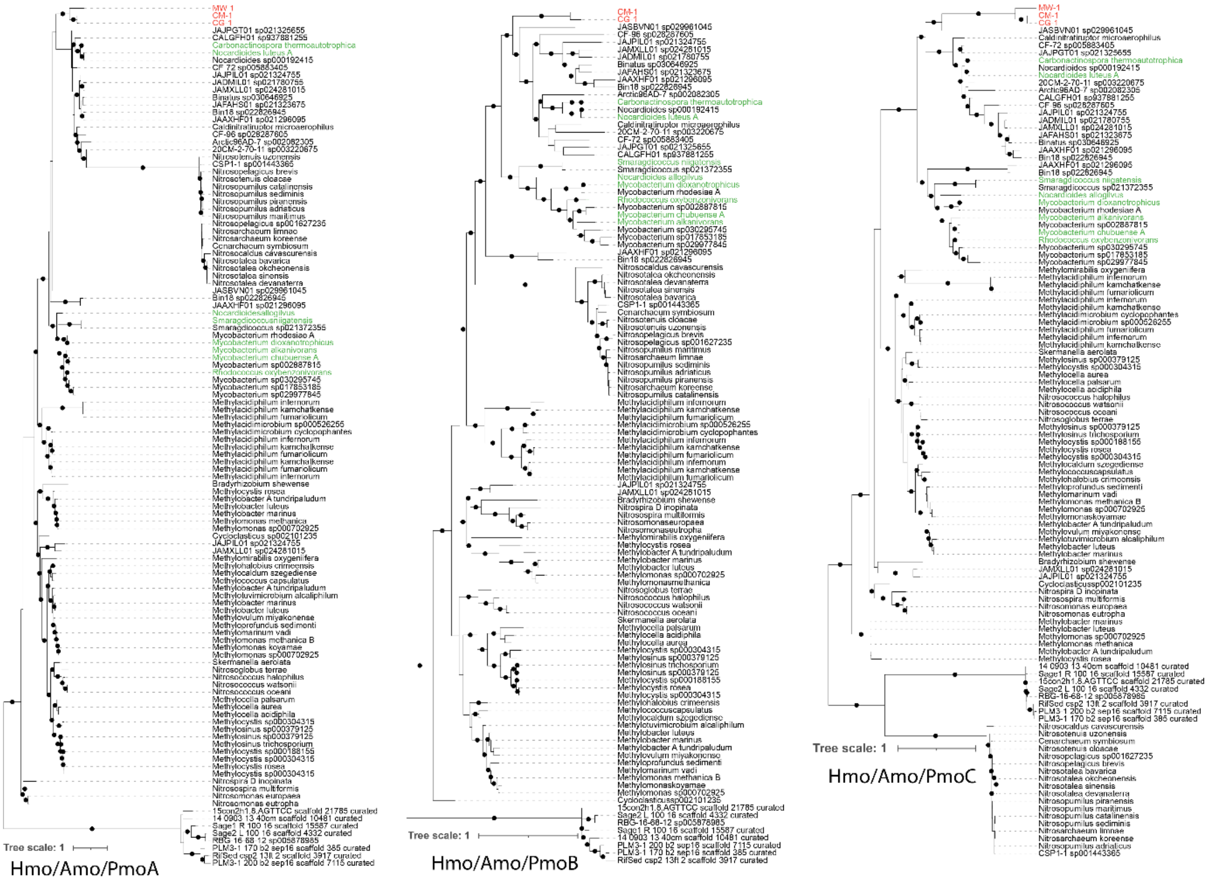

Supplementary Figure 2. Maximum likelihood tree of individual Hmo/Amo/Pmo subunits. All trees were rooted by midpoint. Red text denotes the Hmo subunits from CM-1, CG-1, and MW-1. Green text denotes known Hmo subunits from known hydrocarbon oxidizing bacterium. Bootstrap values were determined by non-parametric bootstrapping of 100 replicates. Black dots indicate ≥75% bootstrap support and the scale bar represent the number of amino acid substitutions per site.

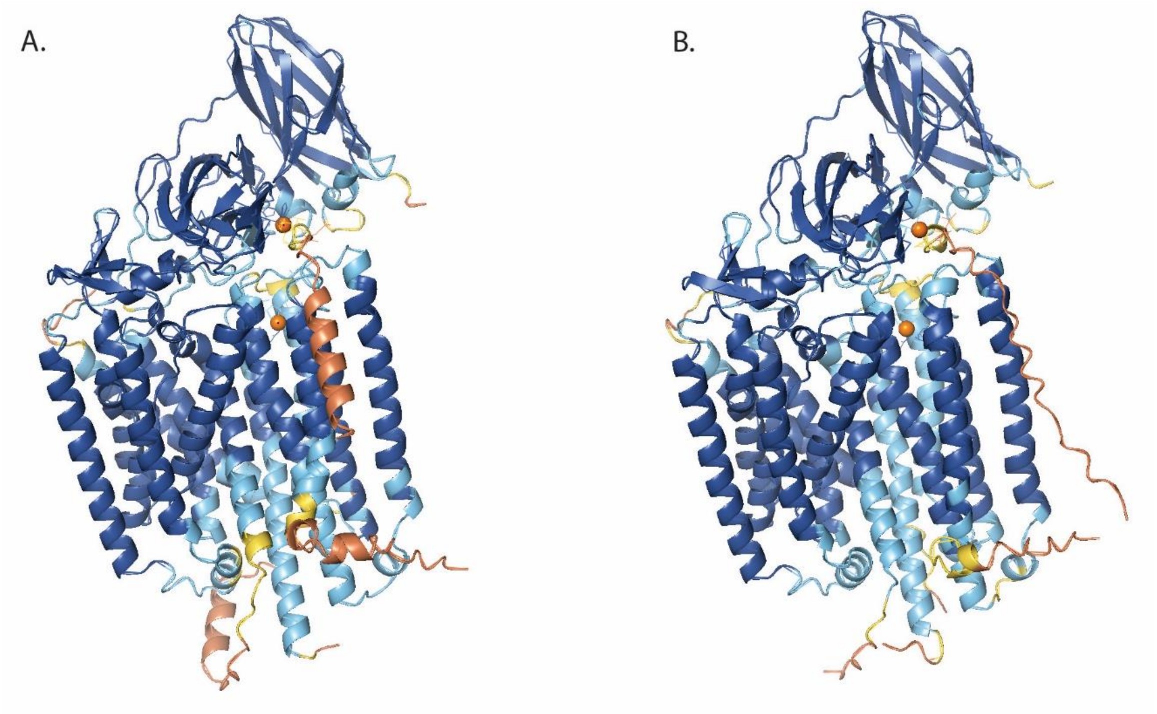

Supplementary Figure 3. Alphafold structures and transplanted copper ligand sites. A, B. CuMMO complexes of CM-1 and CG-1 are shown respectively. Predicted structures were coloured by their pLDDT according to Alphafold colouring scheme[1]. Transplanted copper ligands were embedded into the predicted structure using alphafill[2]. Alphafold pdb files for CM-1 and CG-1 are deposited in Supplementary Data 3 & 4.

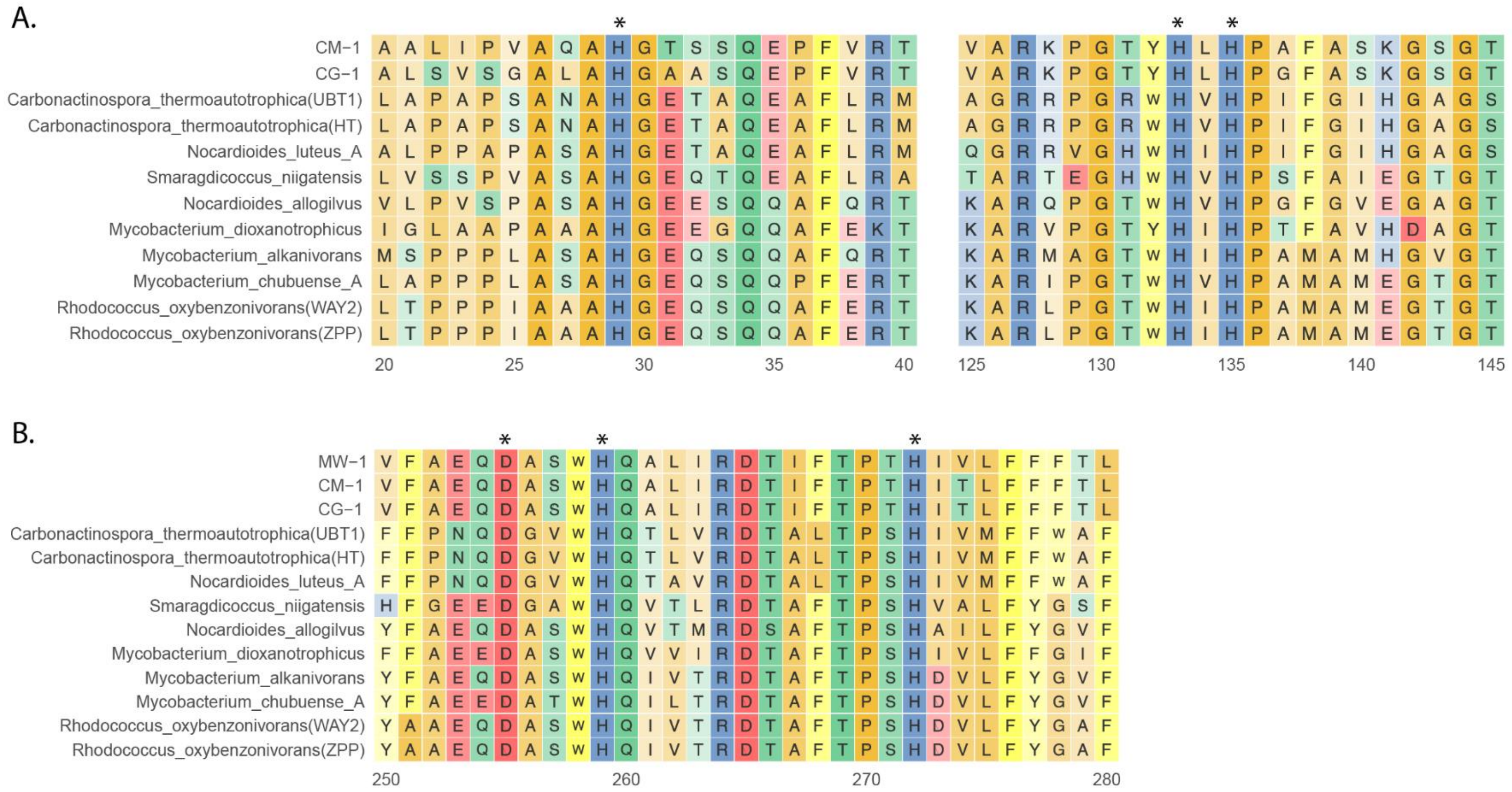

Supplementary Figure 4. Sequence alignments of CuMMO sequences from CM-1, CG-1, MW-1, and known hydrocarbon oxidizers. A. HmoB protein alignments. **B.** HmoC protein alignments. Asterisk denotes conserved residues that are putative copper binding sites.

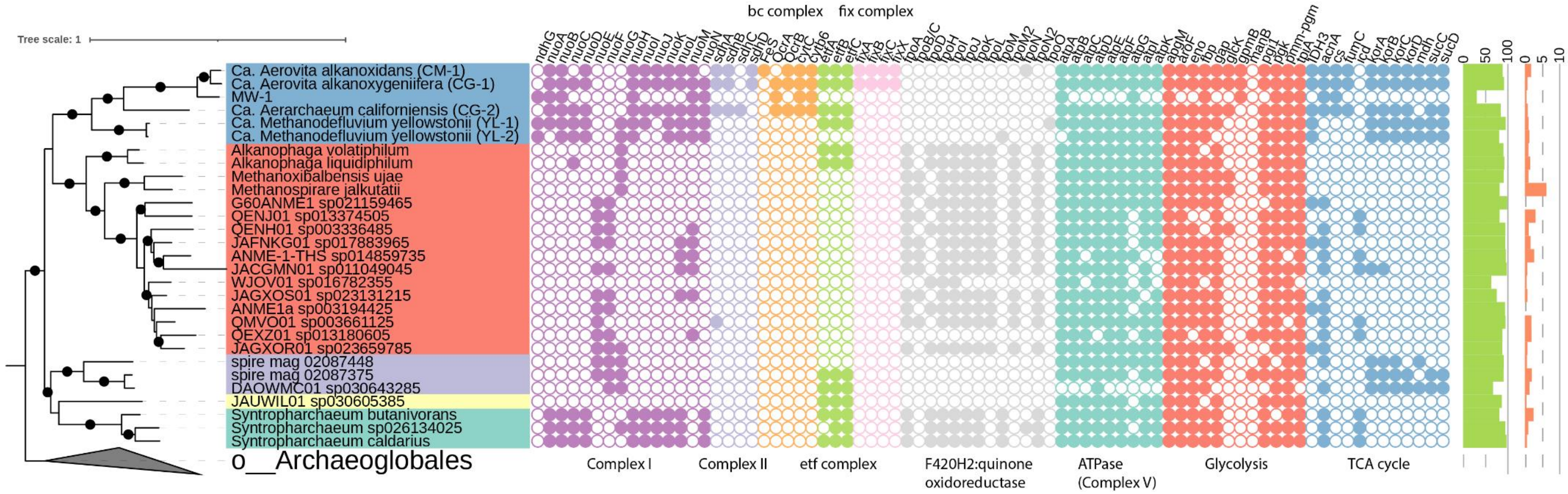

Supplementary Figure 5. Maximum-likelihood tree and additional metabolisms of interests. Genomes were dereplicated to the best species per genera based on CheckM2. Genomes were coloured based on their order level placement, Blue: *Ca.* 'Aerarchaeales'; Red: Alkanophagales; Purple: DAOWMC01; Yellow: JAUWIL01; Green; Syntropharchaeales. Bootstrap values were determined by non-parametric bootstrapping of 100 replicates. Black dots indicate ≤75% bootstrap support and the scale bar represent the number of amino acid substitutions per site. Coloured circles denote the occurrence of genes in the given lineage. See Supplementary Data 5 for detailed annotation information.

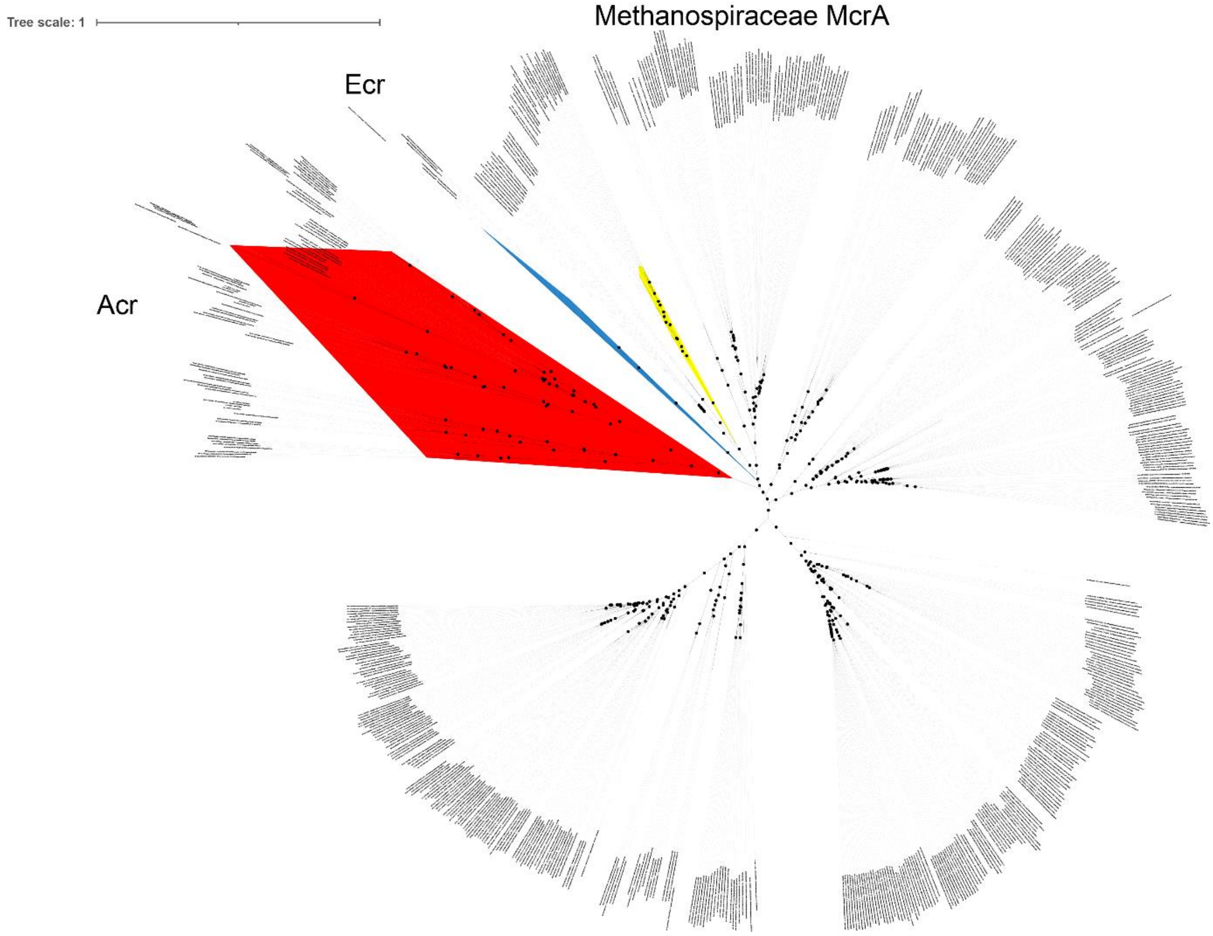

Supplementary Figure 6. Maximum likelihood tree of Alkyl/methyl-coenzyme M reductase subunit A (McrA/AcrA). Bootstrap values were determined by non-parametric bootstrapping of 100 replicates. Black dots indicate ≥75% bootstrap support and the scale bar represent the number of amino acid substitutions per site. Red, blue, and yellow shading denote AcrA, EcrA, and the Methanospiraceae McrA sequences.

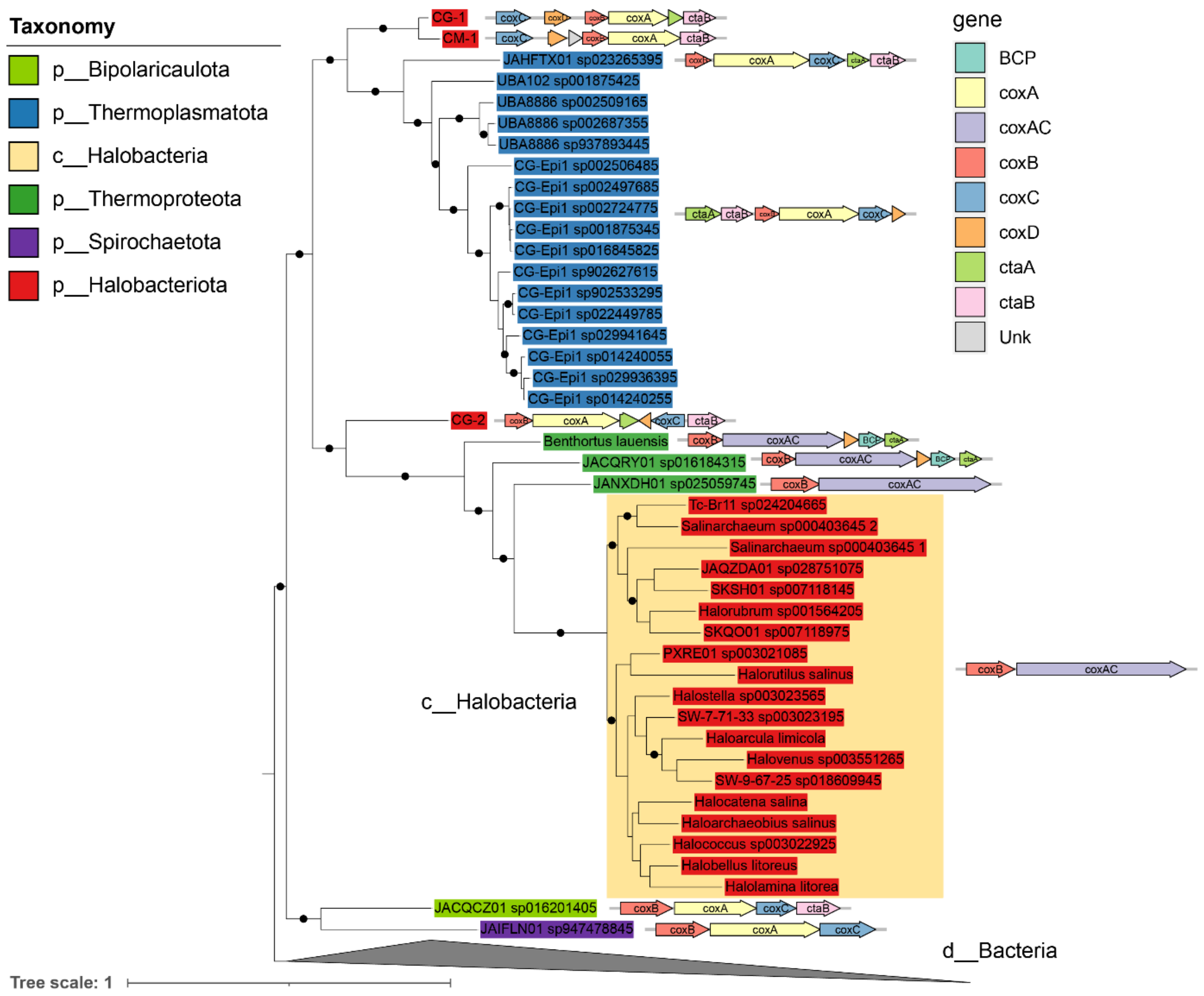

Supplementary Figure 7. Complex IV phylogeny and genetic organisation. The maximum likelihood tree of the coxA subunit of heme copper oxygen reductase shows two independent acquisitions of complex IV are likely within f__CG-2. CG-1 complex IV are shown to be distantly related to the Thermoplasmatota as evident with the phylogeny and the genetic organization. The origins of CG-2 complex IV is unknown. Bootstrap values were determined by non-parametric bootstrapping of 100 replicates. Black dots indicate ≥75% bootstrap support and the scale bar represent the number of amino acid substitutions per site.

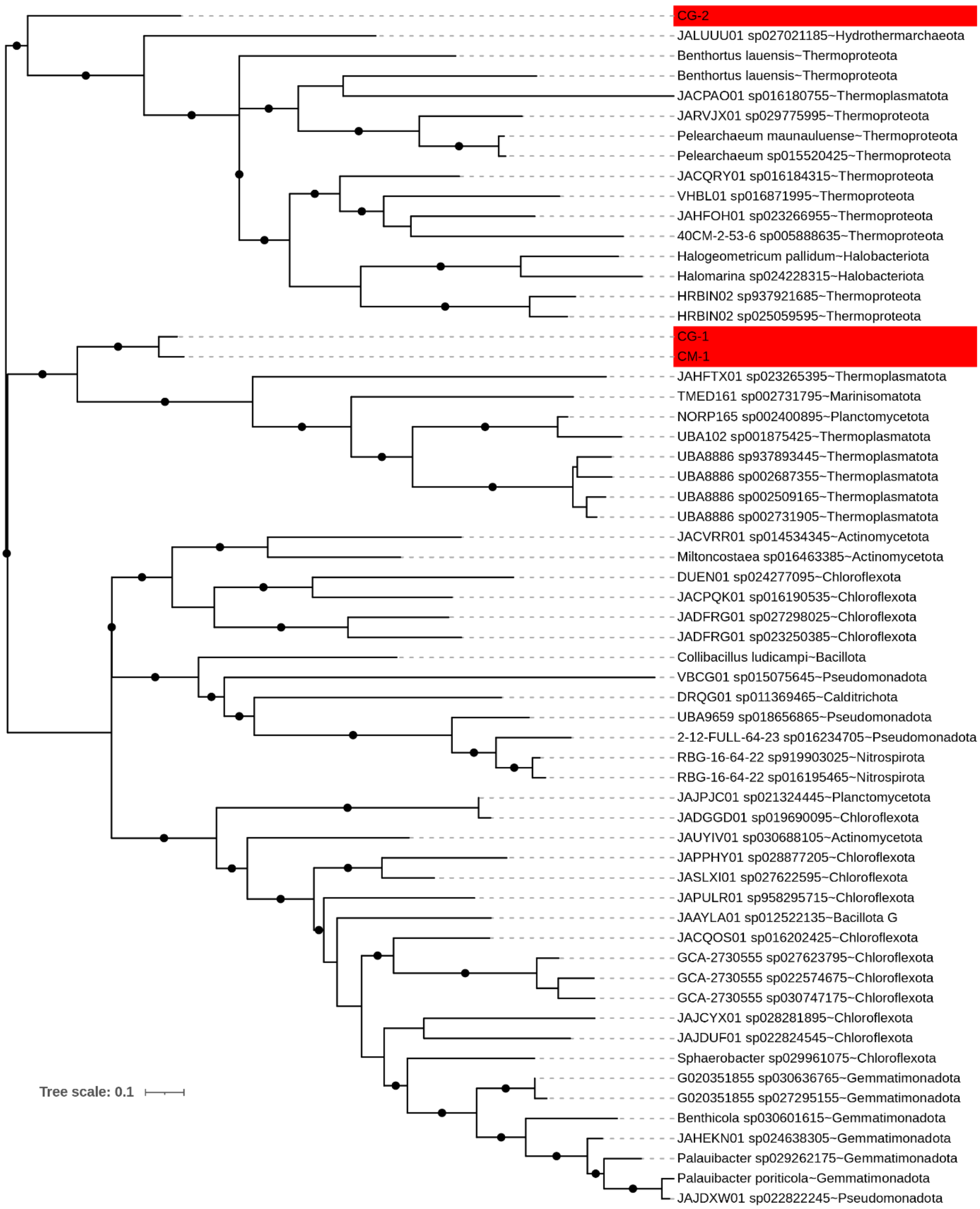

Supplementary Figure 8. Maximum-likelihood phylogenetic tree of cytochrome c oxidase subunit II (coxB). Genes indicated in red denote the *Ca.* 'Aerarchaeaceae' MAGs. Black dots indicate ≥75% bootstrap support and the scale bar represent the number of amino acid substitutions per site.

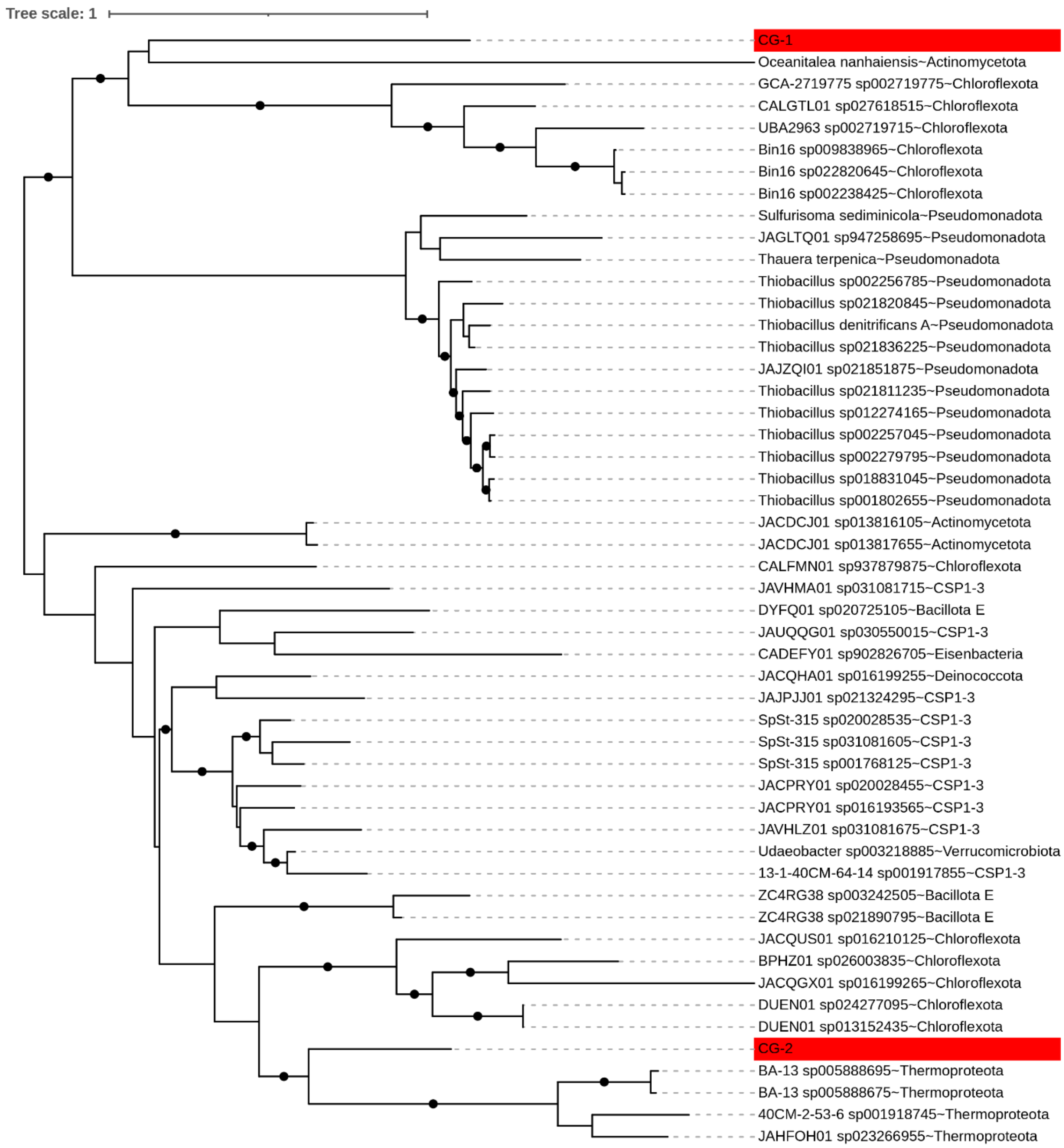

Supplementary Figure 9. Maximum-likelihood phylogenetic tree of heme synthase A (ctaA). Genes indicated in red denote the *Ca.* 'Aerarchaeaceae' MAGs. Black dots indicate ≥75% bootstrap support and the scale bar represent the number of amino acid substitutions per site.

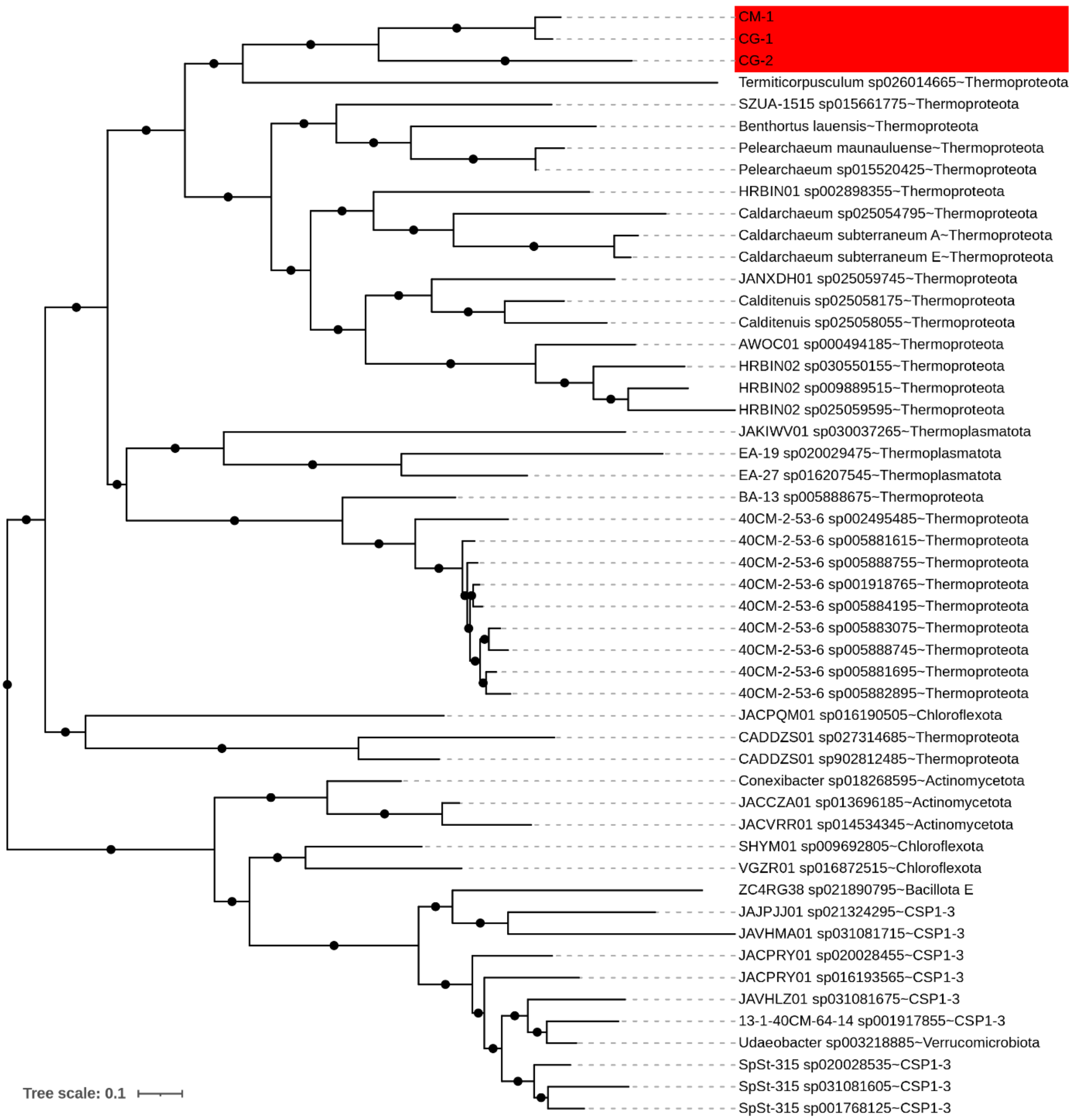

Supplementary Figure 10. Maximum-likelihood phylogenetic tree of heme synthase O (ctaB). Genes indicated in red denote the *Ca.* ‘Aerarchaeaceae’ MAGs. Black dots indicate ≥75% bootstrap support and the scale bar represent the number of amino acid substitutions per site.

Supplementary Note 1

Etymology

Description of the proposed species ‘Candidatus Aerarchaeum californiensis’

Ae.rar.chae.um ca.li.for.ni.en′sis. N.L. neut. n. Aerarchaeum, air-related archaeon; L. adj. californiensis, pertaining to California; N.L. neut. n. Aerarchaeum californiensis, an air-related archaeon from California. This organism is not cultured and is represented by the MAG CG-2, obtained from Californian groundwater samples. The presence of aerobic respiration-related enzymes suggests aerobic metabolism.

Description of the proposed species ‘Candidatus Aerovita alkanoxidans’

Ae.ro.vi’ta al.kan.ox.i′dans. N.L. neut. n. Aerovita, life supported by air; L. neut. adj. alkanoxidans, oxidizing alkanes; N.L. neut. n. Aerovita alkanoxidans, an air-supported organism capable of oxidizing alkanes. This organism is not cultured and is represented by MAG CM-1, with the hydrocarbon monooxygenase (HMO) complex supporting aerobic hydrocarbon oxidation.

Description of the proposed species ‘Candidatus Aerovita alkanoxygeniifera’

Ae.ro.vi’ta al.kan.o.xy.ge.ni.i′fe.ra. N.L. neut. n. Aerovita, life supported by air; N.L. neut. adj. alkanoxygeniifera, bringing oxygen for alkanes; N.L. neut. n. Aerovita alkanoxygeniifera, an air-supported organism associated with alkane oxidation. This organism is not cultured and is represented by the MAG CG-1, distinguished as a separate species within the Aerovita genus.

Description of the proposed family ‘Candidatus Aerarchaeaceae’

Ae.rar.chae.a.ce’ae. N.L. neut. n. Aerarchaeum, a (Candidatus) genus name; -aceae ending to denote a family; N.L. neut. pl. n. Aerarchaeaceae, the (Candidatus) Aerarchaeum family. The description is the same as for the candidate type genus Aerarchaeum. This family also contains the candidate genus Aerovita.

Description of the proposed species ‘Candidatus Methanodefluvium yellowstonii’

Me.tha.no.de.flu’vi.um yel.low.sto′nii. N.L. neut. n. Methanodefluvium, methane-related but methane-loss-associated family; L. gen. n. yellowstonii, of Yellowstone; N.L. neut. n. Methanodefluvium yellowstonii, a methane-related organism from Yellowstone. This organism is not cultured and is represented by MAG YL-1, recovered from sediments in Yellowstone Lake, with a partial loss of the methanogenesis pathway.

Description of the proposed family ‘Candidatus Methanodefluvium’

Me.tha.no.de.flu’vi.um. N.L. neut. n. Methanodefluvium, from methane and defluvium (flowing out), indicating loss of methanogenic function; N.L. neut. n. Methanodefluvium, the methane-associated family with evidence of partial or complete loss of methanogenesis.

Description of the proposed order ‘Candidatus Aerarchaeales’

Ae.rar.chae.a′les. N.L. neut. n. Aerarchaeum, a (Candidatus) genus name; -ales ending to denote an order; N.L. neut. pl. n. Aerarchaeales, the (Candidatus) Aerarchaeum order. The description is the same as for the candidate type genus Aerarchaeum. This order also contains the candidate genus Aerovita and Methanodefluviium.

Supplementary References

1 Jumper, J. *et al.* Highly accurate protein structure prediction with AlphaFold. *nature* **596**, 583-589 (2021).

2 Hekkelman, M. L., de Vries, I., Joosten, R. P. & Perrakis, A. AlphaFill: enriching AlphaFold models with ligands and cofactors. *Nature Methods* **20**, 205-213 (2023).
