# Supplementary material for: Potential for aerobic hydrocarbon oxidation in archaea
Source: Nat Commun. 2025 Oct 16;16:9188. doi: 10.1038/s41467-025-64223-2 (PMC12533140; doi:10.1038/s41467-025-64223-2)
Supplement: Supplementary file 2 — Description of Additional Supplementary Files [file 41467_2025_64223_MOESM2_ESM.pdf]

Description of Additional Supplementary Files:

Supplementary Data 1: MAG completeness and recovery.

Supplementary Data 2: Alphafill transplanted ligand quality metrics.

Supplementary Data 3: CM-1 hmoCAB collabfold pdb file.

Supplementary Data 4: CG-1 hmoCAB collabfold pdb file.

Supplementary Data 5: Metabolic annotation table of Syntropharchaeia MAGs.

Supplementary Data 6: Figure 4 metabolic annotations.

Supplementary Data 7: Amalgamated likelihood estimation table.

Supplementary Data 8: IMNGS relative abundance profiles.

Supplementary Data 9: SRA sandpiper relative abundance profiles.
