# Supplementary material for: Potential for aerobic hydrocarbon oxidation in archaea
Source: Nat Commun. 2025 Oct 16;16:9188. doi: 10.1038/s41467-025-64223-2 (PMC12533140; doi:10.1038/s41467-025-64223-2)
Supplement: Supplementary file 4 — Reporting Summary [file 41467_2025_64223_MOESM4_ESM.pdf]

nature portfolio

Corresponding author(s): Andy Leu and Gene Tyson

Last updated by author(s): Aug 21, 2025

Reporting Summary

Nature Portfolio wishes to improve the reproducibility of the work that we publish. This form provides structure for consistency and transparency in reporting. For further information on Nature Portfolio policies, see our Editorial Policies and the Editorial Policy Checklist.

Statistics

For all statistical analyses, confirm that the following items are present in the figure legend, table legend, main text, or Methods section.

n/a	Confirmed	
☐	☒	The exact sample size (*n*) for each experimental group/condition, given as a discrete number and unit of measurement
☒	☐	A statement on whether measurements were taken from distinct samples or whether the same sample was measured repeatedly
☒	☐	The statistical test(s) used AND whether they are one- or two-sided<br>*Only common tests should be described solely by name; describe more complex techniques in the Methods section.*
☒	☐	A description of all covariates tested
☒	☐	A description of any assumptions or corrections, such as tests of normality and adjustment for multiple comparisons
☒	☐	A full description of the statistical parameters including central tendency (e.g. means) or other basic estimates (e.g. regression coefficient) AND variation (e.g. standard deviation) or associated estimates of uncertainty (e.g. confidence intervals)
☒	☐	For null hypothesis testing, the test statistic (e.g. *F*, *t*, *r*) with confidence intervals, effect sizes, degrees of freedom and *P* value noted<br>*Give P values as exact values whenever suitable.*
☒	☐	For Bayesian analysis, information on the choice of priors and Markov chain Monte Carlo settings
☒	☐	For hierarchical and complex designs, identification of the appropriate level for tests and full reporting of outcomes
☒	☐	Estimates of effect sizes (e.g. Cohen's *d*, Pearson's *r*), indicating how they were calculated

Our web collection on statistics for biologists contains articles on many of the points above.

Software and code

Policy information about availability of computer code

Data collection	Open source software used in this analysis is referenced in Methods: Sandpiper v0.3.0 and GTDB v220.
Data analysis	Open source software used in this analysis is referenced in Methods: SeqPurge v2018_0460, MetaSPAdes v3.10.0, SPAdes v3.10.0, MEGAHIT v1.2.9, BamM v1.7.3, UniteM v0.0.14, aviary v0.2.0, RefineM v0.0.24, GTDB-Tk v2.4.0, CheckM2 v1.0.2, Prokka v1.14.6, DIAMOND v0.9.30.131, KofamScan v1.3.0 (HMMs downloaded 26-04-2024), RPS-BLAST v2.12.0, gggenomes v1.0.1 (R package), Barrnap v0.9, MUSCLE v5.01278, IQ-TREEv2.3.3, FastTree v2.1.11, pargenes v1.2.0, ModelTest-NG v0.1.7, RAxML-NG v1.0.1, ColabFold local v1.5.2, Mol* Viewer v4.17.0, PyMOL v2.5.7, Foldseek v8-ef4e960, AlphaFill v2.1.0, trimAl v1.4.rev15, Treerecs v1.2, ALE v1.0, SingleM v0.18.0, IMNGS Version 1.0 Build 2508, iTOL v6, R v4.3.3, sf v1.0-21.

For manuscripts utilizing custom algorithms or software that are central to the research but not yet described in published literature, software must be made available to editors and reviewers. We strongly encourage code deposition in a community repository (e.g. GitHub). See the Nature Portfolio guidelines for submitting code & software for further information.

Data

Policy information about availability of data

All manuscripts must include a data availability statement. This statement should provide the following information, where applicable:

- Accession codes, unique identifiers, or web links for publicly available datasets
- A description of any restrictions on data availability
- For clinical datasets or third party data, please ensure that the statement adheres to our policy

The MAGs assembled in this study have been deposited in the NCBI database under the accession numbers SAMN45772796 [https://www.ncbi.nlm.nih.gov/biosample/45772796] to SAMN45772801 [https://www.ncbi.nlm.nih.gov/biosample/45772801] under the Bioproject ID PRJNA1197096 [https://www.ncbi.nlm.nih.gov/bioproject/1197096].

Research involving human participants, their data, or biological material

Policy information about studies with human participants or human data. See also policy information about sex, gender (identity/presentation), and sexual orientation and race, ethnicity and racism.

Reporting on sex and gender	This research does not involved human participants, thier data, or biological materia
Reporting on race, ethnicity, or other socially relevant groupings	This research does not involved human participants, thier data, or biological materia
Population characteristics	This research does not involved human participants, thier data, or biological materia
Recruitment	This research does not involved human participants, thier data, or biological materia
Ethics oversight	This research does not involved human participants, thier data, or biological materia

Note that full information on the approval of the study protocol must also be provided in the manuscript.

Field-specific reporting

Please select the one below that is the best fit for your research. If you are not sure, read the appropriate sections before making your selection.

☐ Life sciences ☐ Behavioural & social sciences ☒ Ecological, evolutionary & environmental sciences

For a reference copy of the document with all sections, see nature.com/documents/nr-reporting-summary-flat.pdf

Ecological, evolutionary & environmental sciences study design

All studies must disclose on these points even when the disclosure is negative.

Study description	This study reports the discovery of the first aerobic hydrocarbon-oxidizing archaeon, belonging to a novel order within the Syntropharchaeia. Genomic analyses were performed on six novel MAGs and 23 representative species from the Syntropharchaeia, and the evolutionary history of the novel archaeal order was investigated using gene tree–species tree reconciliation techniques.
Research sample	The analysis included 29 Syntropharchaeia genome sequences, including six novel MAGs recovered in this study. These novel MAGs were assembled from publicly available metagenomes derived from diverse groundwater samples collected across geographically distinct locations.
Sampling strategy	Gene duplication, transfer, loss, and origination events were sampled from 27 branches, representing all internal branches of the Syntropharchaeia species tree. This sample size is the total number of internal branches on the Syntropharchaeia species tree used for gene tree-species tree reconcilition.
Data collection	Metagenomes were downloaded from NCBI sequence read archive database (www.ncbi.nlm.nih.gov). MAGs were downloaded from the GTDB v220 (https://data.gtdb.ecogenomic.org/releases/release220/).
Timing and spatial scale	Collection of data from public repositories was conducted between 2018 to 2024.
Data exclusions	No data have been excluded.
Reproducibility	Not applicable as this was a data analysis study. software and databases used to analyze this data can be accessed in the data availability section and methods section.

Randomization	The genomes were analyzed as a single group. Genes encoded by the MAGs are related by amino acid sequence information and not be any clustering approaches and can thus be considered randomized.
Blinding	The genomes were analyzed as a single group with no priori clustering. Researchers were not blind to the taxonomic placement of the MAGs. This information was not used to infer the phylogenies of the genes and genomes.

Did the study involve field work? ☐ Yes ☒ No

Reporting for specific materials, systems and methods

We require information from authors about some types of materials, experimental systems and methods used in many studies. Here, indicate whether each material, system or method listed is relevant to your study. If you are not sure if a list item applies to your research, read the appropriate section before selecting a response.

Materials & experimental systems

n/a	Involved in the study	
☒	☐	Antibodies
☒	☐	Eukaryotic cell lines
☒	☐	Palaeontology and archaeology
☒	☐	Animals and other organisms
☒	☐	Clinical data
☒	☐	Dual use research of concern
☒	☐	Plants

Methods

n/a	Involved in the study	
☒	☐	ChIP-seq
☒	☐	Flow cytometry
☒	☐	MRI-based neuroimaging

Plants

Seed stocks	No seed stocks were used.
Novel plant genotypes	No plants were used in this study.
Authentication	No plants were used in this study, hence no authentication was used.
